# A composite strategy of genome-wide association study and copy number variation analysis for carcass traits in a Duroc pig population

**DOI:** 10.1186/s12864-022-08804-1

**Published:** 2022-08-13

**Authors:** Rongrong Ding, Zhanwei Zhuang, Yibin Qiu, Xingwang Wang, Jie Wu, Shenping Zhou, Donglin Ruan, Cineng Xu, Linjun Hong, Ting Gu, Enqin Zheng, Gengyuan Cai, Wen Huang, Zhenfang Wu, Jie Yang

**Affiliations:** 1 College of Animal Science and National Engineering Research Center for Breeding Swine Industry, South China Agricultural University, Guangdong, 510642 P.R. China; 2 Guangdong Wens Breeding Swine Technology Co., Ltd, Guangdong, 527439 P.R. China; 3 Lingnan Guangdong Laboratory of Modern Agriculture, Guangzhou, 510642 China; 4 Department of Animal Science, Michigan State University, East Lansing, MI USA

**Keywords:** Pigs, Copy number variation, GWAS, Carcass traits

## Abstract

**Background:**

Carcass traits are important in pig breeding programs for improving pork production. Understanding the genetic variants underlies complex phenotypes can help explain trait variation in pigs. In this study, we integrated a weighted single-step genome-wide association study (wssGWAS) and copy number variation (CNV) analyses to map genetic variations and genes associated with loin muscle area (LMA), loin muscle depth (LMD) and lean meat percentage (LMP) in Duroc pigs.

**Results:**

Firstly, we performed a genome-wide analysis for CNV detection using GeneSeek Porcine SNP50 Bead chip data of 3770 pigs. A total of 11,100 CNVs were detected, which were aggregated by overlapping 695 CNV regions (CNVRs). Next, we investigated CNVs of pigs from the same population by whole-genome resequencing. A genome-wide analysis of 21 pigs revealed 23,856 CNVRs that were further divided into three categories (851 gain, 22,279 loss, and 726 mixed), which covered 190.8 Mb (~ 8.42%) of the pig autosomal genome. Further, the identified CNVRs were used to determine an overall validation rate of 68.5% for the CNV detection accuracy of chip data. CNVR association analyses identified one CNVR associated with LMA, one with LMD and eight with LMP after applying stringent Bonferroni correction. The wssGWAS identified eight, six and five regions explaining more than 1% of the additive genetic variance for LMA, LMD and LMP, respectively. The CNVR analyses and wssGWAS identified five common regions, of which three regions were associated with LMA and two with LMP. Four genes (*DOK7*, *ARAP1*, *ELMO2* and *SLC13A3*) were highlighted as promising candidates according to their function.

**Conclusions:**

We determined an overall validation rate for the CNV detection accuracy of low-density chip data and constructed a genomic CNV map for Duroc pigs using resequencing, thereby proving a value genetic variation resource for pig genome research. Furthermore, our study utilized a composite genetic strategy for complex traits in pigs, which will contribute to the study for elucidating the genetic architecture that may be influenced and regulated by multiple forms of variations.

**Supplementary Information:**

The online version contains supplementary material available at 10.1186/s12864-022-08804-1.

## Background

In recent decades, pork has made up a large share of total worldwide meat production to meet demands for more animal protein products and accommodate growing human consumption [1]. For the pig industry, carcass traits are known to play an essential role in pig breeding programs with the purpose of improving pork production. One of the strategies that can accelerate the genetic progress of these economically important traits is to incorporate of genome-wide association studies (GWASs) results in genomic prediction models, which can improve genomic prediction accuracy [2]. For polygenic quantitative traits, GWAS using high-density single-nucleotide polymorphisms (SNPs) has become a powerful tool to dissect the genetic architecture of complex traits by leveraging linkage disequilibrium (LD) between the causative mutations and common SNP markers [3]. Many studies have focused on this important point and utilized multiple strategies to detect quantitative trait loci (QTLs) and genes for carcass traits, such as the identified QTLs for loin muscle area (LMA), loin muscle depth (LMD) [4], and lean meat percentage (LMP) [5]. GWASs for these traits in pigs were performed based on animals that were genotyped and phenotyped. However, much genealogical information of animal breeding farms has been ignored in the innovation of GWAS methodology. To make full use of genealogical information and phenotypes of genotyped and non-genotyped animals, a GWAS method under the single-step genomic best linear unbiased prediction (ssGBLUP) framework was developed and referred to as a “weighted single-step GWAS” (wssGWAS) [6]. This powerful method has been used in pigs [7] to detect QTLs and genes associated with economically important traits. These traits are polygenic quantitative traits and may be influenced and regulated by multiple forms of genetic variations that consequently are expressed as different phenotypes of specific traits. However, the associations between copy number variation (CNV) and LMA, LMD and LMP have not been widely investigated. CNVs can be defined as segments of DNA including gains and losses of genomic sequence (over a length of > 1 kb) that differ from the reference genome [8]. It has been shown that CNVs play important roles in regulating gene expression, consequently affecting specific phenotypes in pigs [9]. Regarding the detection of CNVs in pigs, SNP arrays were the popular platform and several studies have focused on multiple pig breeds to identify existing CNVs and further define the number of CNV regions (CNVRs) using SNPs Bead chip [10]. With the rapidly decreasing costs of next generation sequencing (NGS) and the possibility of discovering a multitude of variant classes, NGS-based CNV detection have been used in domestic animals, including pigs [11]. Moreover, improvement in NGS technology that increase accuracy would significantly facilitate the discovery of CNVs by using small sample size in comparison with that used in SNP array-based studies [11].

The main objective of this study was to identify genetic variations and genes associated with carcass traits including LMA, LMD and LMP, by integrating a wssGWAS and CNV analyses in approximately 3770 Duroc pigs that were genotyped with the GeneSeek Porcine SNP50 Bead chip. Herein, we also investigated CNVs of 21 Duroc pigs from this population by NGS data and then, we used the resequencing pigs to determine an overall validation rate for the CNV detection accuracy of chip data. These two approaches (CNVR and SNP based GWAS) were complementary to each other and such a utilization of composite genetic strategy for complex traits in pigs provides valuable insights into elucidating the genetic architecture that may be influenced and regulated by multiple forms of variations.

## Results

### Genome-wide detection of CNVs and CNVRs

**Fig. 1:**
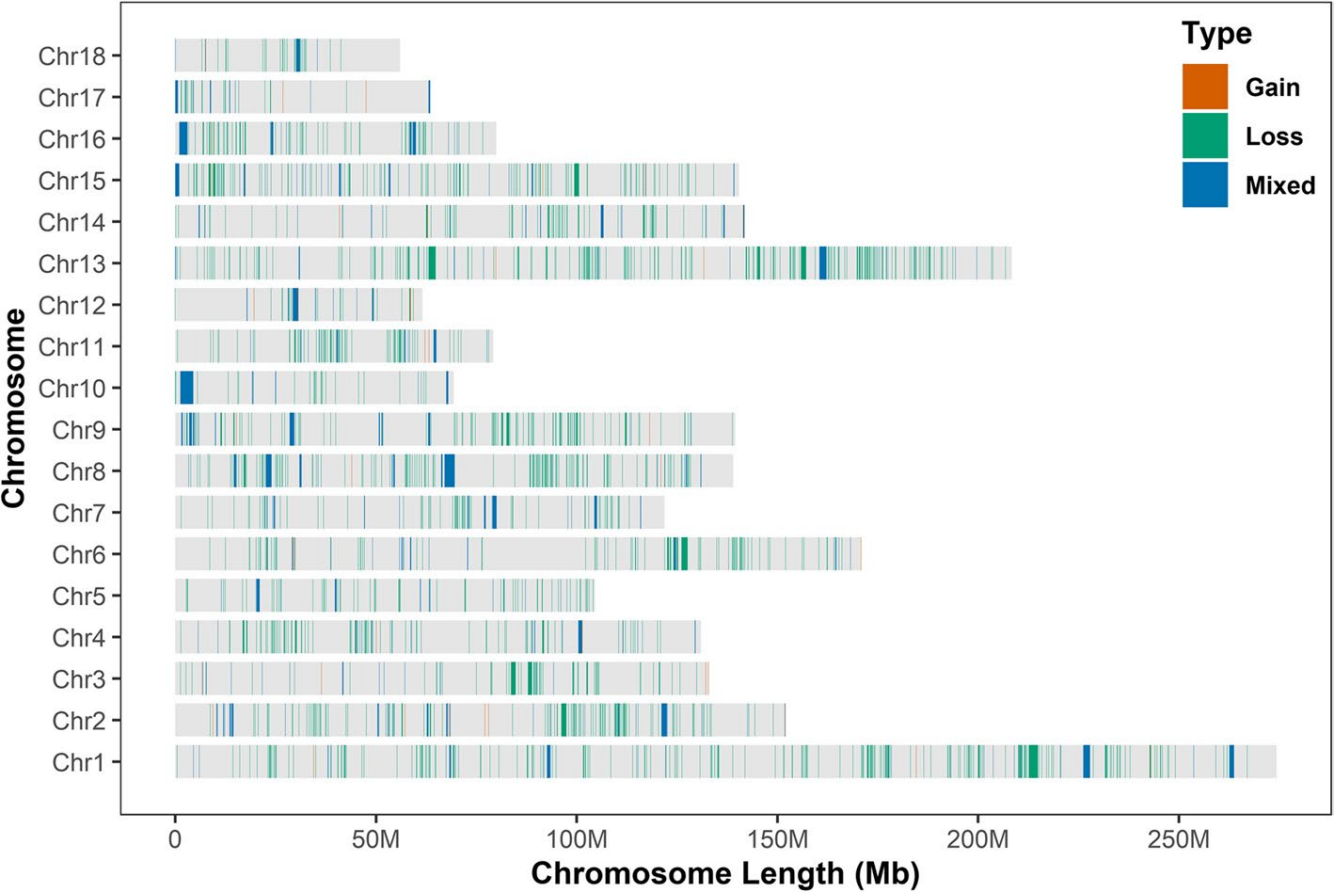
The overall CNVR map for Duroc pigs in the 18 autosomes. Three types of CNVR are identified, including gain (red), Loss (green), and Mixed (blue). Y-axis values are autosomes, and X-axis values are chromosome position in Mb

**Fig. 2:**
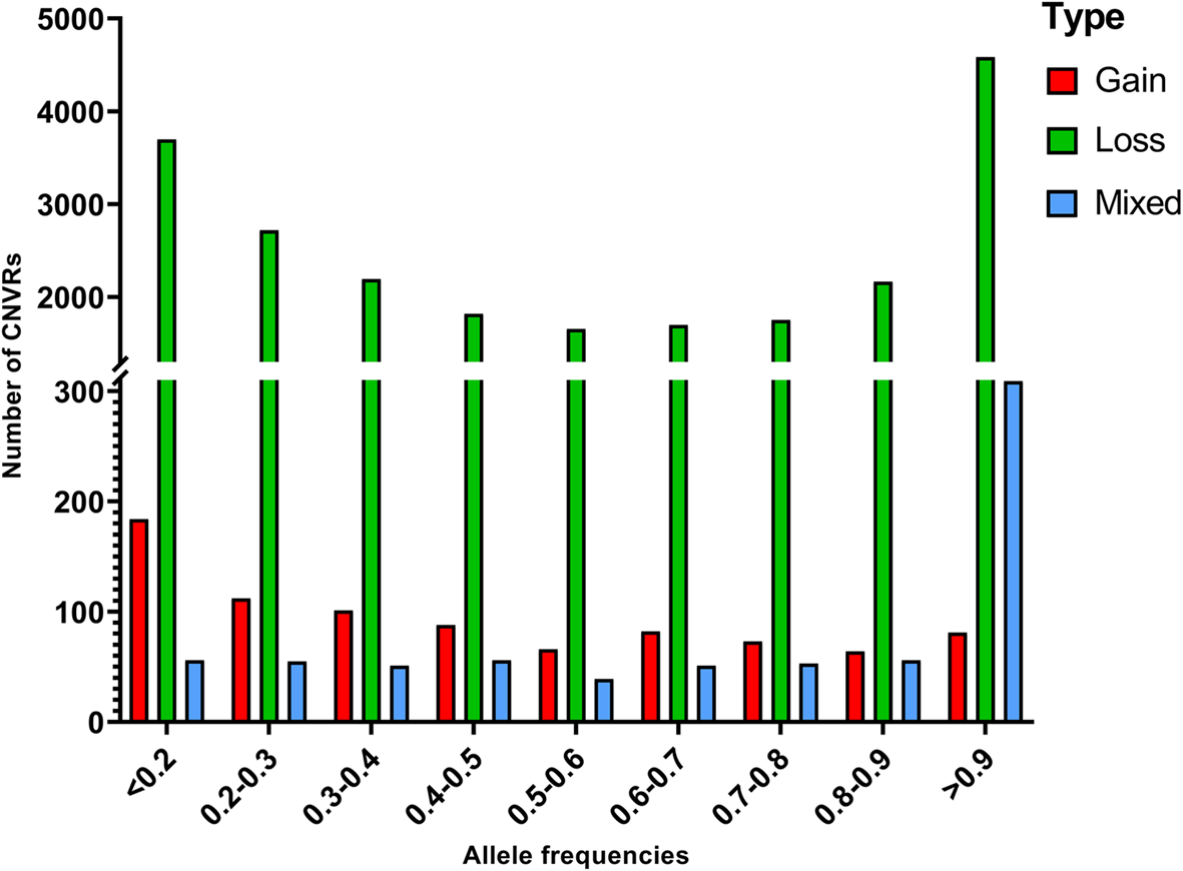
The allele frequencies of variants in the resequencing Duroc pigs

For 50 K SNP array, 3261 animals and 38,894 SNPs were remained to identify CNV events after applying stringent filtering criteria. A total of 11,100 CNV events (8093 gains and 3007 losses) were detected, which were aggregated by overlapping 695 CNVRs. The length of these CNVRs ranged from 11.80 kb to 4.25 Mb (Additional file 1: Table S1). Among these CNVRs, 273 corresponded to CNV gains, 301 to losses, and 121 were mixed. These CNVRs covered approximately 174.43 Mb of the pig autosomal genome and corresponded to 7.7% of the genome sequence (Additional file 2: Table S2). For NGS data, A genome-wide analysis of 21 pigs revealed 23,856 CNVRs (Fig. 1) that were further divided into three categories, i.e., gain (*n* = 851), loss (*n* = 22,279), and mixed (*n* = 726) CNVRs, covering 190.8 Mb (~ 8.42%) of the pig autosomal genome (Table 1). Among these CNVRs, 96.89% (23,114) of CNVRs’ segment length were less 50 kb. The proportion of CNVRs varied from 5.5% (Sus scrofa chromosome 17, SSC17) to 11.94% (SSC16) across the 18 autosomes. We calculated the allele frequencies of the CNVRs in the 21 re-sequencing Duroc pigs. Results showed the detecting frequency for loss was higher than that for gain and mixed CNVRs (Fig. 2).

**Table 1:** Chromosome distribution of all 23,856 CNVRs detected in the porcine genome (*Sscrofa* 11.1 reference genome assembly) by next generation sequencing data

Chr	Chr length (bp)	CNVR count	Total CNVR length (bp)	Average size (bp)	Percentage (%)
1	274330532	2187	24465457	11186.77	8.92
2	151935994	1570	15064612	9595.29	9.92
3	132848913	1347	7921458	5880.82	5.96
4	130910915	1528	9733311	6369.97	7.44
5	104526007	1163	6678547	5742.52	6.39
6	170843587	1474	10681625	7246.69	6.25
7	121844099	1405	7533446	5361.88	6.18
8	138966237	1499	16186457	10798.17	11.65
9	139512083	1462	13185580	9018.86	9.45
10	69359453	1025	6445133	6287.93	9.29
11	79169978	1245	7090443	5695.13	8.96
12	61602749	863	4177951	4841.19	6.78
13	208334590	1913	22275135	11644.09	10.69
14	141755446	1330	8144486	6123.67	5.75
15	140412725	1386	14849166	10713.68	10.58
16	79944280	992	9542775	9619.73	11.94
17	63494081	822	3493138	4249.56	5.50
18	55982971	645	3326,348	5157.13	5.94
Total	2265774640	23856	190795068	7997.78	8.42

### Validation of CNVRs detected in 50 K SNP array

**Fig. 3:**
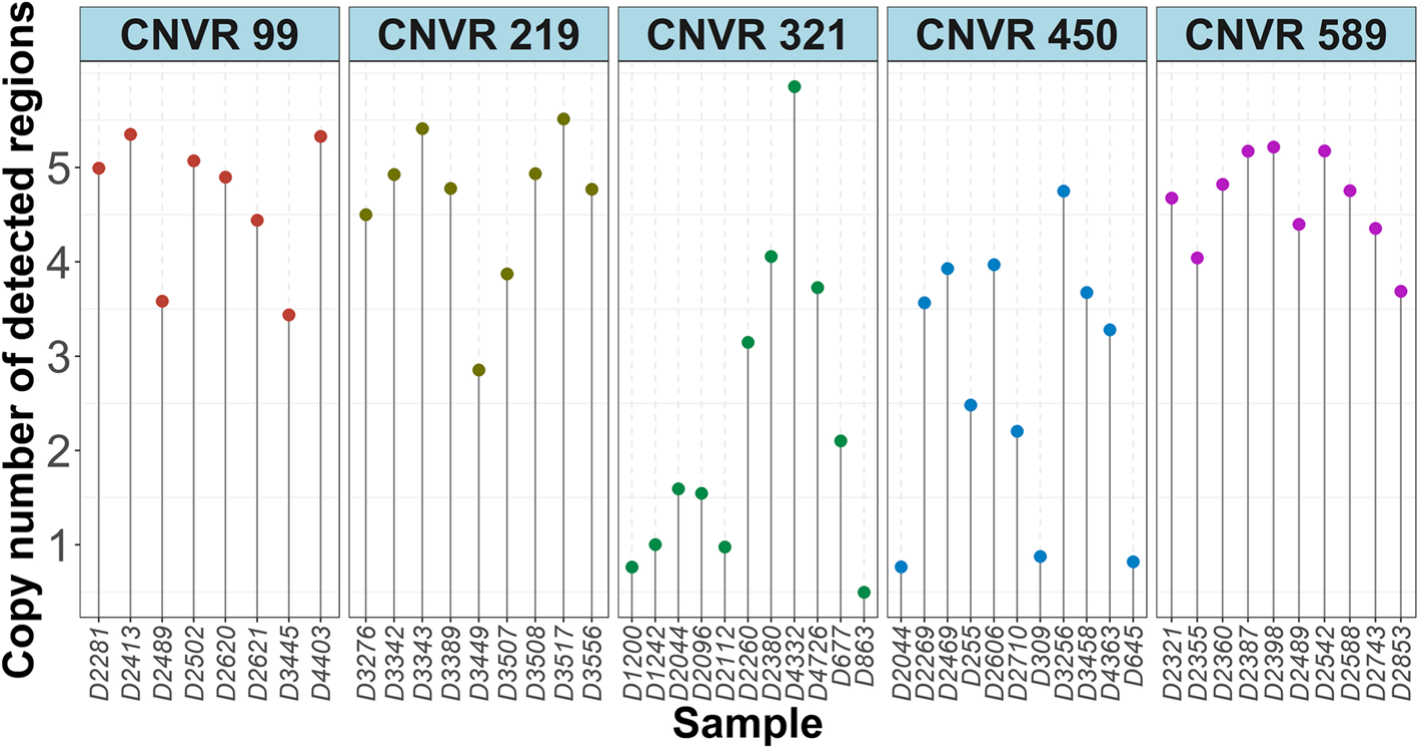
The results of qPCR validation in selected CNVRs detected in 50 K SNP array. The x-axis represents the tested sample ID. The y-axis represents different copy number. Values of approximately 2 were considered normal. A value of 3 or more and a value of 1 or less represented gain and loss statuses, respectively

We investigated CNVs of 20 pigs from the same population by whole-genome resequencing to determine an overall validation rate of 68.5% for the CNV detection accuracy of chip data. 50 of the 73 CNVRs detected in 50 K SNP array of the 20 pigs were validated in NGS-based CNVRs results (Additional file 3: Table S3). Furthermore, we randomly selected six CNVRs (CNVR ID: CNVR 99, 219, 241, 321, 450, and 589) that co-localized with *ADAP1*, *FANCA*, *GPR153*, *LRPAP1*, *MCF2L*, and *CFAP46* genes, respectively. Five of these CNVRs (CNVR 99, 219, 321, 450, and 589) were successfully validated (Fig. 3).

### CNVR-based association analyses for carcass traits

**Fig. 4:**
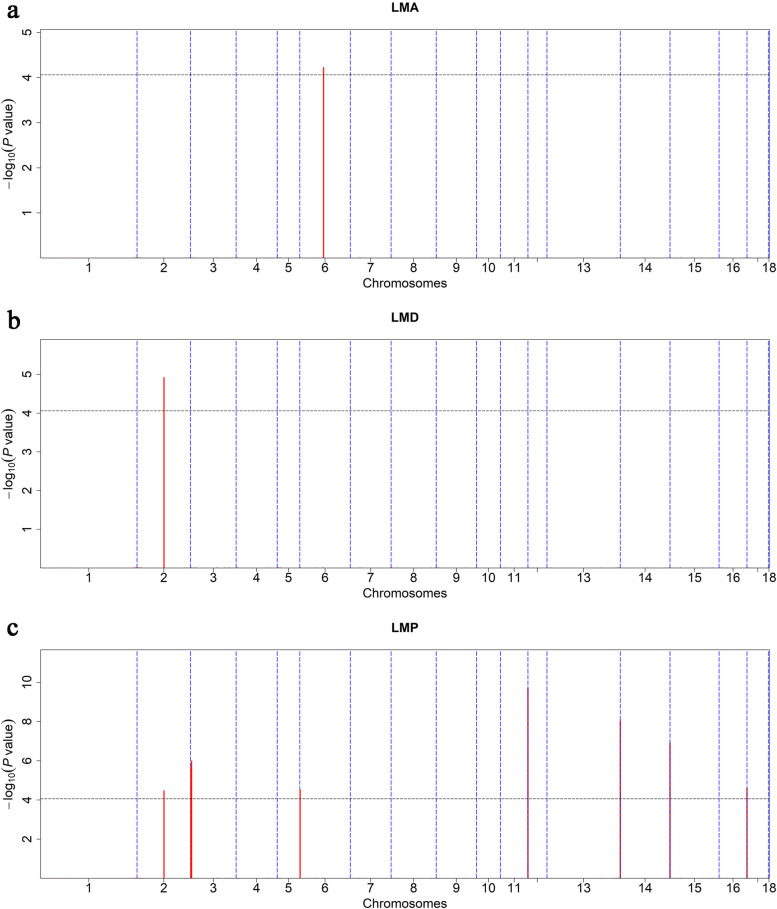
Manhattan plots of CNVR-based association analyses for LMA (**a**), LMD (**b**) and LMP (**c**) in Duroc pigs

After quality control for the identified CNVRs in 50 K SNP array prior to conducting association analyses, 117 CNVRs remained to perform association tests for LMA, LMD and LMP in this Duroc pigs. A stringent criterion of Bonferroni correction was adopted for the association tests for LMA, LMD and LMP to determine the genome-wide significant *P* value threshold, which was defined as 8.55E-05 (0.05/117). As shown in Table 2 and Fig. 4, we detected 10 CNVRs associated with carcass traits, of which one (ID: CNVR241) was associated with LMA (Fig. 4a), one (ID: CNVR71) was associated with LMD (Fig. 4b) and eight (ID: CNVR71, CNVR99, CNVR101, CNVR219, CNVR450, CNVR534, CNVR589, and CNVR654) were associated with LMP (Fig. 4c). For LMA, the significant CNVR241 was a mixed-type CNVR located on SSC6, 66.98 – 67.38 Mb, and covering approximately 393 kb of the genome sequence, together with 11 genes. Interestingly, CNVR27, located on SSC2, 76.01 – 76.54 Mb and covering 521 kb of the genome sequence, was found to be associated with both LMD and LMP traits in this Duroc pigs. Within the CNVR27, we detected 14 protein-coding genes that potentially contribute to both traits. For LMP, the most significant CNVR, CNVR450, was located on SSC11, 77.14 – 78.78 Mb. Seventeen genes including *COL4A2*, *RAB20*, *NAXD, CARS2*, *ING1*, *ANKRD10*, *ARHGEF7*, *TUBGCP3*, *ATP11A*, *MCF2L*, *PCID2*, *CUL4A*, *GRTP1*, *DCUN1D2*, *TMCO3*, *TFDP1*, and *ATP4B* were detected and highlighted as potential candidates for LMP. Other prominent CNVRs associated with LMP were also analyzed, and genes within these CNVRs were detected according to the porcine reference genome annotation from the Ensembl database (Table 2).

**Table 2:** Significant CNVRs associated with LMA, LMD and LMP in Duroc pigs

Traits	Chr	CNVR ID	Type	Start (bp)	End (bp)	Length (bp)	*P*-value	Genes
LMA	6	CNVR241	Mixed	66986351	67379563	393212	6.01E-05	*KCNAB2, CHD5, RNF207, HES3, GPR153, ACOT7, HES2, ESPN, PLEKHG5, TNFRSF25, NOL9*
LMD	2	CNVR71	Mixed	76013400	76535013	521613	1.21E-05	*GADD45B, LMNB2, TMPRSS9, SPPL2B, LSM7, LINGO3, PEAK3, DOT1L, AMH, JSRP1, AP3D1, IZUMO4, MOB3A, MKNK2*
LMP	2	CNVR71	Mixed	76013400	76535013	521613	3.39E-05	*GADD45B, LMNB2, TMPRSS9, SPPL2B, LSM7, LINGO3, PEAK3, DOT1L, AMH, JSRP1, AP3D1, IZUMO4, MOB3A, MKNK2*
	3	CNVR99	Mixed	162027	2026411	1864384	2.21E-06	*FAM20C, PDGFA, PRKAR1B, DNAAF5, SUN1, COX19, CYP2W1, C7orf50, GPER1, ZFAND2A, UNCX, MICALL2, INTS1, MAFK, TMEM184A, PSMG3, ELFN1, MAD1L1, SNX8, EIF3B, CHST12, GRIFIN, LFNG, TTYH3, IQCE, BRAT1, GNA12*
	3	CNVR101	Mixed	2457661	3480462	1022801	1.02E-06	*SDK1*
	6	CNVR219	Mixed	51842	1347980	1296138	3.05E-05	*PRDM7, GAS8, DEF8, TCF25, SPIRE2, FANCA, ZNF276, VPS9D1, CDK10, DPEP1, SPG7, ANKRD11, CDH15, ACSF3, CBFA2T3, PABPN1L, GALNS, APRT, PIEZO1, CTU2, RNF166, SNAI3, MVD, CYBA, IL17C, ZC3H18, ZFPM1, ZNF469*
	11	CNVR450	Mixed	77144460	78780052	1635592	1.92E-10	*COL4A2, RAB20, NAXD, CARS2, ING1, ANKRD10, ARHGEF7, TUBGCP3, ATP11A, MCF2L, PCID2, CUL4A, GRTP1, DCUN1D2, TMCO3, TFDP1, ATP4B*
	13	CNVR534	Mixed	206578011	208240759	1662748	9.15E-09	*RRP1B, RRP1, AGPAT3, AIRE, PFKL, CFAP410, TRPM2, TSPEAR, UBE2G2, PTTG1IP, ADARB1, COL18A1, SLC19A1*
	14	CNVR589	Mixed	139524158	141719266	2195108	1.22E-07	*TCERG1L, BNIP3, JAKMIP3, DPYSL4, STK32C, LRRC27, PWWP2B, INPP5A, NKX6-2, CFAP46, ADGRA1, KNDC1, ADAM8, TUBGCP2, CALY, MTG1, SCART1, CYP2E1*
	16	CNVR654	Mixed	78550922	79365542	814620	2.49E-05	*IRX4, NDUFS6, MRPL36, LPCAT1, SLC6A3, CLPTM1L, SLC6A18, SLC6A19, SLC12A7*

### Weighted single-step genome-wide association study

**Fig. 5:**
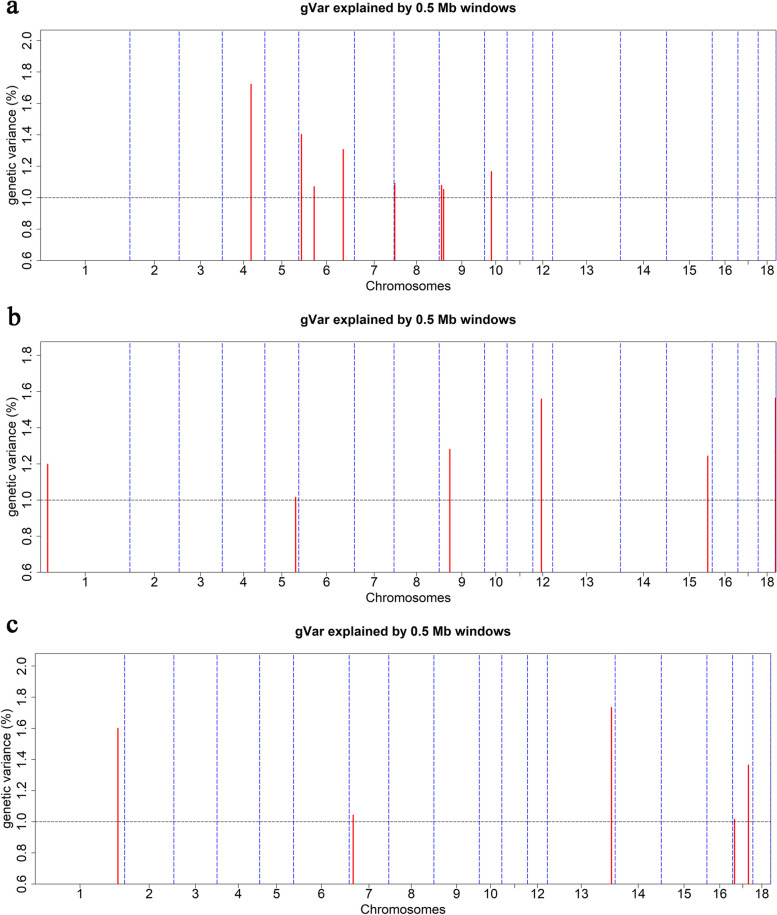
Manhattan plots of wssGWAS for LMA (**a**), LMD (**b**) and LMP (**c**) in Duroc pigs. The results of the weighted single-step GWAS are represented by the proportion of additive genetic variance explained by 0.5 Mb windows. Windows that each explain > 1% of the additive genetic variance are highlighted

The estimated heritability based on pedigree information for LMA, LMD, and LMP was 0.48 ± 0.06, 0.43 ± 0.07, and 0.60 ± 0.07, respectively (Additional file 4: Table S4). Nineteen genomic windows were detected each explaining > 1% of the additive genetic variance for the three traits on SSC1, 4, 5, 6, 7, 8, 9, 10, 12, 13, 15, 17, and 18 (Table 3 and Fig. 5). The explained genetic variance of SNPs within the significant windows for LMA, LMD, and LMP were list in Additional file 5: Table S5, Additional file 6: Table S6, and Additional file 7: Table S7, respectively. Moreover, these significant genomic windows explained 9.89%, 7.86%, and 6.76% of the additive genetic variance for LMA, LMD and LMP, respectively. Of the 19 windows, eight were found to be associated with LMA (Fig. 5a). The most significant window (ID: 4_88), explaining 1.72% of the additive genetic variance for LMA, was located at 88.52 – 88.84 Mb and contained the genes *OLFML2B*, *ATF6*, *DUSP12*, and *FCRLB*. For LMD, six windows (Fig. 5b) were associated with the trait. The most significant windows (ID: 12_26 and 18_53) both explained 1.56% of the additive genetic variance for LMD and were located at 26.33 – 26.83 Mb and 53.36 – 53.82 Mb, respectively. The analysis of the association with LMP identified five windows (Fig. 5c) that were located on SSC1, 7, 13, and 17. Window 13_197 explained 1.73% of the additive genetic variance for LMP and seven related genes were highlighted as candidates for LMP.

**Table 3:** Identification of genes based on the additive genetic variance (gVar) explained by 0.5 Mb windows of the adjacent SNPs

Traits	Chr	window ID	Start position (bp)	End position (bp)	nSNPs	gVar (%)	Candidate genes
LMA	4	4_88	88528079	88837975	7	1.72	*OLFML2B, ATF6, DUSP12, FCRLB*
	6	**6_7**	7944029	8429029	24	1.40	*/*
	6	6_47	47252470	47728980	9	1.07	*CATSPERG, PSMD8, GGN, SPRED3, FAM98C, RASGRP4, MAP4K1, ACTN4, HNRNPL, CAPN12, ECH1, RINL, SIRT2, NFKBIB, CCER2, MRPS12*
	6	6_136	136587431	137008535	12	1.31	*ST6GALNAC3*
	8	**8_2**	2113824	2538785	18	1.09	*DOK7, ADRA2C, HMX1*
	9	**9_7**	7165800	7643984	11	1.08	*PDE2A, ARAP1, ATG16L2, FCHSD2*
	9	9_13	13689451	14174146	23	1.05	*/*
	10	10_21	21285879	21768391	13	1.17	*ATP6V1G3, PTPRC*
LMD	1	1_21	21550279	22025895	14	1.20	*PHACTR2, FUCA2, PEX3, ADAT2, AIG1*
	5	5_94	94705788	95188437	17	1.01	*/*
	9	9_32	32672891	33160648	7	1.28	*CEP126, CFAP300, YAP1, BIRC3, TMEM123*
	12	12_26	26333018	26829227	9	1.56	*PPP1R9B, SGCA, COL1A1, TMEM92, XYLT2, MRPL27, EME1, LRRC59, ACSF2, CHAD, RSAD1, MYCBPAP, EPN3, SPATA20*
	15	15_126	126576898	127069740	30	1.24	*DOCK10, NYAP2*
	18	18_53	53366998	53823227	16	1.56	*SUGCT*
LMP	1	1_253	253922492	254393867	10	1.60	*PRPF4, RNF183, WDR31, BSPRY, HDHD3, ALAD, C9orf43, RGS3*
	7	7_12	12342523	12829394	11	1.04	*ATXN1*
	13	**13_197**	197092574	197585514	23	1.73	*TMEM50B, DNAJC28, GART, SON, DONSON, CRYZL1, ITSN1*
	17	17_5	5673866	6148397	14	1.02	*PCM1, ASAH1*
	17	**17_48**	48234481	48714245	13	1.36	*NCOA5, CDH22, SLC35C2, ELMO2, OCSTAMP, SLC13A3*

### Common regions identified by the wssGWAS and CNVR analyses

We identified common regions between the wssGWAS and CNVR analyses on SSC6 (7.94 – 8.42 Mb), SSC8 (1.87 – 3.85 Mb), and SSC9 (7.16 – 7.64 Mb) that were associated with LMA, and on SSC13 (197.09 – 197.58 Mb) and SSC17 (48.23 – 48.71 Mb) that were associated with LMP (Table 4). Protein-coding genes within the common regions were highlighted as candidates for the traits analyzed. Moreover, the windows on SSC6 and SSC13 were in detection CNVRs; the windows on SSC8 and SSC9 were in mixed CNVRs; and the window on SSC17 was in a duplication CNVR.

**Table 4:** Common regions between CNVRs and the significant windows detected by wssGWAS

Traits	Chr	window ID	CNVR ID	Start position (bp)^a^	End position (bp)	Type	Length (bp)
LMA	6	6_7	CNVR_222	8252860	8393964	Loss	141104
	8	8_2	CNVR_321	1876229	3858083	Mixed	1981854
	9	9_7	CNVR_365	7165800	7526785	Mixed	360985
LMP	13	13_197	CNVR_526	197092574	197570477	Loss	477903
	17	17_48	CNVR_675	48351314	48427404	Gain	76090

^a^ as of “Start position” columns are the characterization of the CNVRs

### KEGG and GO enrichment analyses

The KEGG and GO enrichment analyses of the 219 genes from all carcass traits revealed seven significant pathways (such as metabolic pathways, collecting duct acid secretion, MAPK signaling pathway and ubiquitin mediated proteolysis) and 75 significant GO terms. Detailed information on the significant pathways and terms is listed in Additional file 8: Table S8.

## Discussion

### General discussions about the genetic detection of LMA, LMD and LMP traits

In this study, the combination of a region-based GWAS with CNVR analyses improved the detection efficiency of genomic regions and genes associated with LMA, LMD and LMP and consequently identified additional genetic variants and genes. LMA and LMD, which are regarded as carcass traits, play essential roles in the determination of LMP and other growth traits [12]. Previously, we utilized a meta-analysis of GWASs to identify QTLs associated with LMA and LMD in two Duroc pig populations and successfully detected remarkable QTLs for the traits analyzed [4]. However, previous genetic studies conducted for LMA, LMD and LMP utilized a classic GWAS based on single marker regression in genotyped animals. This inadequate use of genealogical information and genetic variants may result in ignoring other important loci in the genome. By using a wssGWAS and CNV analyses, the present study identified missing QTLs that were not detected previously, thus overcoming the aforementioned deficiencies to some extent. The SNPs within identified regions will be useful for the genetic improvement of carcass traits by allowing the associated SNPs to be assigned with higher weights in genomic selection. In this study, we removed 578 CNVRs applying stringent filtering criteria to reduce the false-positive rates of results and a stringent criterion of Bonferroni correction was adopted for the association tests for LMA, LMD and LMP to determine the genome-wide significant *P* value threshold. This led to a limited number CNVRs were used in the association analyses. Although the findings of identified CNVRs may help elucidate the genetic architecture of carcass traits that may be influenced and regulated by multiple forms of variations, the translation of the CNVs in these regions into applications in the pig industry will not be as direct as that of SNPs in the genomic selection for LMA, LMD and LMP [13]. These variants, however, may have value in increasing selection accuracy by genotyping the CNVR types according to the copy number value and stetting the CNVRs represented in a specific genotype [14], and then incorporated them as prior biological information into genomic models such as genomic feature BLUP approach (GFBLUP) [15]. Moreover, there are some limitations of this study that should be pointed out. In the procedure of wssGWAS, we calculated the SNP weights followed default recommendations made by the developers of the BLUPF90 software packages. Such weighting methodology may overestimate some regions while shrinking others to 0. Recently, the developers proposed several new procedures for calculating SNP weights in wssGBLUP that can be effective in improving both the accuracy of genomic estimated breeding values (GEBV) and SNP effects [16]. In the future, these advanced approaches for calculation of SNP weights should be used to reach maximum predictively of SNP effects.

### CNV detection accuracy and comparison with previous studies of porcine CNVRs

The validation results of CNVRs detected in 50 K SNP array implied that the CNV detection accuracy of chip data in the present study were relatively low, since the overall validation rate between resequencing data and microarray data in human was 83.5%, which utilized a population-level of 266 paired-end Illumina data to call CNVs [17]. In this study, among the CNVRs detected in resequencing data, 96.89% (23,114) of CNVRs’ segment length were less 50 kb while the value was 9.93% (69) of 50 K SNP array, implying that the CNVRs detected by microarray data was sparsely distributed on the genome. Although using resequencing data to call CNVs improved the CNVR map density in pig genome, it seemed that genome resequencing of large-scale individuals and higher sequence coverage have the potential to increase the CNV detection accuracy both for low coverage resequencing and microarray data, or using third-generation sequencing-based data may be the better strategy for CNV calling [18]. Thus, stringent quality control should be applied for CNVRs while conducting subsequent association analyses. To determine whether the CNVRs identified in our study overlapped with those reported in previous studies, we compared our results in Duroc pigs to those CNVRs identified in several different swine studies [8, 10, 19–22]. All CNVRs identified in other studies were converted to *Sscrofa* 11.1 genome assembly using the liftOver tool. In total, 56.69% (394/695) of the CNVRs in the present study overlapped with the merged CNVRs of previous studies by at least 1 bp (Additional file 9: Table S9). For instance, Chen et al. [21] detected 565 CNVRs in 1693 pigs from 18 diverse populations using the Porcine SNP60 Bead chip and PennCNV algorithms and consequently, 47 CNVRs overlapped with this study with a total CNVR length of 4,668,695 bp. Wang et al. [20] performed CNV discovery in 12 pigs from the Asian wild boar population, six Chinese indigenous breeds, and Yorkshire and Landrace commercial pigs using a custom-designed 1 M array comparative genomic hybridization (CGH) and detected 758 CNVRs, covering 47.43 Mb of the pig genome. Among these 758 CNVRs, we identified 19 CNVRs that overlapped with the reported CNVRs in this study with a total CNVR length of 699,128 bp. Zhang et al. [22] performed genome-wide CNV detection on 46 pigs including Suhai, Minzhu, and Large White based on NGS data and 11,173 CNVRs were detected. Among these CNVRs, we identified 323 CNVRs that overlapped with the reported CNVRs in this study. Recent reports regarding the detection and characterization of CNVRs include a study evaluating the traits of piglets born alive in a Duroc pig population [8]. Using Porcine SNP80 Bead chip data and the PennCNV algorithm, the study identified 425 CNVRs covering 197 Mb of the pig genome from 3520 Duroc pigs, and 56 CNVRs with a total CNVR length of 8,534,850 bp overlapped with the CNVRs in our study. Specifically, NGS is capable of revealing far more novel data, and efforts to uncover differences between SNP array data and next-generation sequencing data in terms of detecting CNVs have largely emphasized the examination of the number of SNPs and the density and influence of different calling platforms [11, 19]. Therefore, different CNVR map patterns in pigs were observed after comparison with the results of the aforementioned studies using different genotyping platforms. In this study, we used a relatively low-density GeneSeek Porcine SNP50 Bead chip on 3770 animals to detect CNV events, and the detection efficiency of this study was sufficient compared to that of a previous study (sample size = 3520 Duroc pigs) that used a Porcine SNP80 Bead chip (CNVR count in our study vs. previous study = 695 vs. 425) [8]. Copy number variation is a major component of genomic variation and is considered a promising source for some economically important traits in multiple species, such as rock pigeons (*Columba livia*) and pigs [9, 23]. Numerous populations have been genotyped with SNP chips or NGS panels for CNV identification. However, overlapping CNVRs detected in this way have been limited due to the relatively low sample size of analyzed populations and potential breed-specific characteristics [8]. On the other hand, CNV detection studies targeting diverse pig breeds and using different platforms provide important complementary data to the CNV map of the pig genome. We also investigated CNVs of 21 pigs from the same population by whole-genome resequencing to reveal 23,856 CNVRs, covering 8.42% of the pig autosomal genome. The NGS-based CNVRs results provide a high supplement density for the high-resolution map of copy number variation in the porcine genome in comparison with that using SNP array. With the development and cost of high-throughput sequencing, we believe that there will be more studies using large-scale whole-genome resequencing data to uncover copy number variations underlying traits of interest in pig.

### Genomic regions and candidate genes reveal the complexity of the genetic architecture of carcass traits in pigs

Copy number variation has been considered a major source of genomic variation, and multiple CNVR-based association analyses have been conducted to provide evidence for the impact of CNV on phenotypes in pigs [10, 19]. For LMA, the candidate gene *ACOT7*, located in CNVR241, encodes a member of the acyl coenzyme family, and the encoded protein hydrolyzes the CoA thioester of palmitoyl-CoA and other long-chain fatty acids [24, 25]. A previous meta-analysis of GWASs revealed that the *ACOT7* gene is associated with the metabolism and transport of fatty acids or lipids in the *longissimus* muscle in pigs [26], and is therefore involved in *longissimus* muscle development. The *GADD45B* and *JSRP1* genes are mapped to the CNVR71 region (SSC2: 76.01 – 76.54 Mb) and were found to be associated with LMD and LMP traits in pigs. The *GADD45B* gene is a member of a group of genes whose transcript levels are increased following stressful growth arrest conditions and treatment with DNA-damaging agents. The functions of the *GADD45B* gene include the regulation of growth and apoptosis [27], and *GADD45B* has been suggested to be overexpressed in beef *longissimus* thoracis muscles [28]. The *JSRP1* gene encodes a protein that is involved in excitation–contraction coupling at the sarcoplasmic reticulum, and *JSRP1* interacts with *CACNA1S*, *CACNB1*, and calsequestrin to help regulate calcium influx and efflux in skeletal muscle [29]. In addition, the *SDK1* gene, located on the CNVR101 region, and the *PTTG1IP* gene, located on the CNVR534 region, were identified to be associated with LMP. The *SDK1* gene encodes a protein that is a member of the immunoglobulin superfamily. Previous GWASs have shown that *SDK1* is a candidate gene for pork meat quality [30, 31]; however, the mechanism of *SDK1* gene involvement in muscle development has not been clearly defined. The *PTTG1IP* gene encodes a single-pass type I integral membrane protein, which binds to pituitary tumor-transforming 1 protein (*PTTG1*). A recent report regarding transcriptome analyses of genes revealed that the *PTTG1IP* gene is involved in muscle development and was also found to be alternatively spliced among the muscle tissues in chickens [32]. Therefore, it is reasonable to regard *PTTG1IP* as a candidate gene for muscle development in pigs, and it might also be a candidate gene for meat content.

Furthermore, the wssGWAS for LMA, LMD and LMP traits detected 19 genomic windows. Six genes identified in common regions between the wssGWAS and CNVR analyses were highlighted as promising candidates according to their function. For LMA, we highlighted the *DOK7* gene, located on SSC8 (1.87 – 3.85 Mb) and the *ARAP1* gene, located on SSC9 (7.16 – 7.64 Mb). The *DOK7* gene encodes a protein that is essential for neuromuscular synaptogenesis and functions in aneural activation of muscle-specific receptor kinase, which is required for postsynaptic differentiation. The literature does not identify any link between LMA and the *DOK7* gene, as it has rarely been investigated in muscle in pigs or other domestic animals. The *ARAP1* gene plays a role in encoding ARF-GAP, Rho-GAP, ankyrin repeat and pleckstrin homology domain-containing protein 1 [33]. Further studies are needed to clarify the role of these genes in LMA. For LMP, we highlighted the *ELMO2* and *SLC13A3* genes, located on SSC17 (48.23 – 48.71 Mb). The protein encoded by the *ELMO2* gene interacts with the dedicator of cytokinesis 1 protein. The *ELMO2* gene plays a role in the phagocytosis of apoptotic cells and in cell migration and has been implicated in the regulation of Rac1 and Akt activation [34]. The *SLC13A3* gene encodes a high-affinity protein that plays an important role in the processing of citrate by the kidneys. A previous study has shown that the *ELMO2* and *SLC13A3* genes may also play a role in type 2 diabetes. Although these genes have not been reported to be directly related to lean meat content, genes with pleiotropic effects on complex traits may be responsible in this case [35]. For instance, the combined effect of a gene duplication (CNV) and a splice mutation in the *KIT* gene causes dominant white coat color in pigs [36]. However, it has been reported that these mutations have pleiotropic effects on hematopoiesis [36]. Loin muscle area, LMD and LMP are typical polygenic quantitative traits, and many candidate genes have been highlighted in the pig genome. In this study, we proposed several genes as promising candidates for these traits, and these findings will further advance our understanding of the genetic mechanisms of complex traits in pigs.

## Conclusion

This study investigated the CNVs of pigs and provided a high supplement density for the high-resolution map of copy number variation in the porcine genome by using NGS-based and SNP array data. Our results showed an overall validation rate of 68.5% for the CNV detection accuracy of chip data in comparison with that using NGS data. The functions of genes containing unique CNVRs are related to the carcass traits of pigs. From this, we have identified some candidate genes. Such a utilization of composite genetic strategy for complex traits in pigs provides valuable insights into elucidating the genetic architecture that may be influenced and regulated by multiple forms of variations.

## Methods

### Ethics statement

The animals and experimental methods used in this study are following the guidelines of the Ministry of Agriculture of China. The ethics committee of South China Agriculture University (SCAU) (Guangzhou, China) approved this study (Approval number SCAU#0017). All methods are reported in accordance with ARRIVE guidelines (https://arriveguidelines.org).

### Animals, phenotypes and pedigree information

The experimental animals used in this study consisted of 3941 American Duroc pigs (2440 males and 1501 females) that were born from 2013 to 2017 and were raised on two farms of Wen’s Foodstuffs Group Co., Ltd. (Guangdong, China). Among the 3941 pigs, 3770 were genotyped and phenotyped, and 171 pigs were phenotyped but non-genotyped. All pigs sustained uniform feeding conditions and received fine fodder during the fattening period from 30 to 100 kg live weight, as previously described [4]. The pigs were measured for carcass traits with the following methods: LMA, LMD and LMP were collected from the 10^th^-rib to 11^th^-rib when the pigs weighted 100 ± 5 kg by an Aloka 500 V SSD B ultrasound (Corometrics Medical Systems, USA). This machine employs a diagnostic ultrasound system and transducers to acquire high-resolution images. The LMD and LMA were obtained as described by the Canadian Centre for Swine Improvement (http://www.ccsi.ca/Reports/Reports_2007/Update_of_weight_adjustment_factors_for_fat_and_lean_depth.pdf) and our previous paper [4]. The LMP were calculated using the formula as following described [12]:

$$LMP\left(\mathrm{\%}\right)=61.21920-0.77665*BF+0.15239*LMD$$

where BF is the backfat thickness and LMD is the loin muscle depth. Pedigree information was newly added in this study. The pedigree dataset contained genealogical information for all pigs and the completed pedigree of these individuals can be traced back to 4 generations, with 5679 pigs in the full pedigree. In this study, we reported the new information of the results of NGS data and GWAS with CNV compared with previous papers [4, 14], and the details of explanation were described below.

### SNP genotyping and Re-sequencing

Among the 3941 phenotyped Duroc pigs, 3770 animals (131 pigs belonged to the first generation of the genealogical structure were both genotyped and phenotyped) were genotyped using a GeneSeek Porcine SNP50 Bead chip, which contained 50,703 SNPs. The genotype dataset was converted from *Sus scrofa* genome 10.2 to build *Sus scrofa* genome 11.1. Quality control procedures were performed using PLINK v1.07 software [37] with the following criteria: individual call rate > 95%, SNP call rate > 99%, minor allele frequency > 1% and *P* > 10^–6^ for the Hardy–Weinberg equilibrium test. Pigs that met the criteria above remained in the study. SNPs located on the sex chromosomes and unmapped chromosomes were removed.

To further elucidation of the CNVs in Duroc pigs, we performed a detailed characterization of CNVs by investigating the whole genome sequencing data. In brief, a total of 21 samples were randomly selected from the mentioned above 131 pigs of this population and were sequenced on an Illumina HiSeq2000 platform at Novogene (Bejing, China) with 150 bp paired-end reads and an average depth of ~ 10 × . Raw Illumina reads were processed to remove adapter and low-quality sequences. The paired-end reads were aligned to pig reference genome (Sus sacrofa 11.1) by BWA (version 0.7.15) [38] with default parameters. SAMTools Tools (version 1.3.1) [39] and Picard Tools v.2.7.1 (http://broadinstitute.github.io/picard/) were used for data sorting and duplicates marked, respectively.

### CNV and CNVR detection in 50 K SNP array

PennCNV software [40] was used to identify CNVs by incorporating log R Ratio (LRR) and B allele frequency (BAF), which were automatically computed by GenomeStudio software v2.0 from the signal intensity files of the SNP data. The population frequency of the B allele (PFB) file was constructed from the signal files using the compile_pbf.pl routine provided in the PennCNV software. A wave adjustment procedure for genomic waves was also conducted using the -gcmodel option in PennCNV to eliminate the impact of genomic waves on the CNV calling procedure. Raw CNVs that met the criteria of samples with LRR < 0.3, BAF drift < 0.01, GC wave factor of LRR < 0.05, consecutive SNPs ≥ 3, and CNV length ≥ 10 kb were retained for subsequent CNVR definition. Thus, we used bedtools software v2.26.0 [41] to merge CNVs with at least 1 bp overlap in all samples to define the CNVR. The CNVRuler software v1.3.3.2 [42] was used to define three types of CNVR: loss, gain and mixed (gains and losses occurring in the same region) as described in our previous study [14]. We used in-house script to genotype CNVRs in this Duroc pig population into “ + / + ”, “ ± ”, “-/-”, and CNVRs with the frequencies large than 0.5% were remained to conduct CNVR-based association analysis for LMA, LMD and LMP.

### CNV and CNVR detection in NGS data

To generate a maximally sensitive set of copy number variants (CNVs) in the Duroc samples, we carried out CNV calling for each sample using two read depth (RD)-based tools, namely, CNVnator (version 0.4.1) [43], Control-FREEC (version 11.6) [44], and two discordant read pair (RP)-based and split read (SR)-based tools, namely, DELLY (version 0.8.7) [45] and Smoove (version 0.2.6) [46]. For CNVnator, the suggested ratio of the mean reading depth signal to its standard deviation was ~ 4 to 5. Thus, we calculated statistics for a wide ranges of bin sizes (100 to 1000 bps, with 100 bp increments) for 21 samples (Additional file 10: Table S10) using the -eval option in the CNVnator, and the final bin size was then selected for each sample within 400 ~ 800 bps. Additionally, to select the calls with the highest confidence, calls with a q0 ≥ 0.5 were removed, q0 refers to the fraction of reads with a mapping quality of zero in the called CNV. For Control-FREEC, a breakpoint threshold was set to 0.6 to increase sensitivity and obtain more predicted CNVs, a coefficient of variation of 0.1 was used in the analysis (the suggested threshold was 0.05 to 0.1 [47]). For the detection of CNV (deletions or duplications) using the RP and SR based methods, DELLY and Lumpy-based [48] tool Smoove were used with default parameters. For Smoove, Duphold (version 0.2.3) [46] annotations were added for each call. Here, deletions with DHFFC < 0.7 and duplications with DHBFC > 1.3 were retained to further reduce redundancy and obtain high confidence CNVs, DHFFC refers to the fold-change for the variant depth relative to flanking regions and DHBFC describes the fold-change for the variant depth relative to bins in the genome with similar GC-content. Deletions and duplications identified by the four CNV callers were merged with ‘mergeSVcallers’ (https://github.com/zeeev/mergeSVcallers), as described in previous study [17].

### Validation of CNVRs detected in 50 K SNP array

Among the 21 re-sequenced pigs, 20 samples were also genotyped by 50 K SNP array and one sample was not. Thus, this one sample was not used in the validation step. In order to determine an overall validation rate for the CNV detection accuracy of chip data, we extracted the CNVs detected in PennCNV for the same 20 re-sequenced (the average sequence coverage per sample was 12.1 ×) and compared them with the results of four whole genome sequencing (WGS)-based callers, successful validation was determined by at least one call in the WGS-based callers overlaps with the CNVs detected in PennCNVs (at least 1 bp overlap between them). Besides, six CNVRs identified in 50 K SNP array were randomly selected to conduct real-time quantitative polymerase chain reaction (qPCR) to validate the detection accuracy by PennCNV and the qPCR reaction was performed as described in our previous paper [14]. The qPCR primers and probes sequence information for specific regions of CNVRs within the genes were listed in Additional file 11: Table S11. *GCG* gene was selected as the reference locus because of its highly conserved among pigs and exists as a single copy in the reference genome.

### CNVR-based association analysis

A linear mixed model model was employed to conduct a CNVR-based association analysis for LMA, LMD and LMP using GEMMA software [49]. Genomic relationship matrix (GRM) based on SNP dataset was generated using GEMMA software. The statistical linear mixed model for GWAS is described as follows:

$$\mathbf{y}=\mathbf{W}\mathrm{\alpha }+\mathbf{X}\upbeta +u+\upvarepsilon$$

where y is an n × 1 vector of phenotypes; ***W*** is a matrix of covariate (i.e., farms, sex and age and a column of 1 s); α is a vector of corresponding coefficients that includes the intercept; *X* is the vector of CNVR marker genotypes; β represents the corresponding effect of the CNVR; *u* refers to an n × 1 vector of random effects, with *u* ~ MVNn(0, λ τ^−1^** K**), and ε is the vector of random residuals, with ε ~ MVNn(0, τ^−1^*In*). τ^−1^ is the variance of the residual errors; ***K*** is GRM and λ represents the ratio between the two variance components; and ***I*** is the identity matrix, and n refers to the number of pigs. Bonferroni correction was applied to determine the genome-wide significance thresholds, which were defined as 0.05/N, where N is the number of filtered CNVRs for the American Duroc pigs.

### Weighted single-step genome-wide association study

The ssGBLUP framework proposed by Wang et al. [6] has been used to perform wssGWASs. A wssGWAS makes full use of the genealogical information and phenotypes of genotyped and non-genotyped animals in one step. Variance component estimation of each trait was estimated using the AI-REML module in BLUPF90 software prior to conducting the wssGWAS. BLUPF90 family software [50] was used to conduct the wssGWAS using a mixed model for single-trait analysis as described:

$$Y={\varvec{W}}b+{\varvec{Z}}a+e$$

where $Y$ is the vector of phenotypic values; b is the vector of fixed effects, including birth year (5 levels), sex (2 levels), and farms (2 levels); $a$ is the vector of additive genetic effects; **W** is the incidence matrix of fixed effects for relating phenotypes; **Z** refers to the incidence matrix of random effects, and $e$ is the vector of residuals. $a$ and $e$ were assumed to be

$$a \sim N\left(0,{\varvec{H}}{\sigma }_{a}^{2}\right), e \sim N(0,{\varvec{I}}{\sigma }_{e}^{2})$$

where ${\sigma }_{a}^{2}$ and ${\sigma }_{e}^{2}$ are additive genetic variance and residual variance, respectively. ***H*** is a blend of matrices that combined the pedigree and the genomic relationship matrix and ***I*** denotes the identity matrix. The inverse of matrix ***H*** is as follows:

$${H}^{-1}={{\varvec{A}}}^{-1}+\left[\begin{array}{cc}0& 0\\ 0& {{\varvec{G}}}^{-1}-{{\varvec{A}}}_{22}^{-1}\end{array}\right]$$

where ${{\varvec{A}}}_{22}^{-1}$ is the inverse matrix of the numerator relationship matrix for genotyped animals and ${{\varvec{A}}}^{-1}$ is the inverse of the relationship matrix based on pedigree. ${{\varvec{G}}}^{-1}$ is the inverse matrix of the genomic relationship matrix. The matrix ***G*** was constructed as previously described [51]:

$${\varvec{G}}=\frac{{\varvec{M}}{\varvec{D}}{\varvec{M}}\mathbf{^{\prime}}}{{\sum }_{i=1}^{m}2{p}_{i}\left(1-{p}_{i}\right)}$$

where ***M*** is an incidence matrix of the SNP genotype (aa = 0, Aa = 1 and AA = 2), ***D*** refers to a diagonal matrix of weights for SNP variance, *m* is the number of SNPs and ${p}_{i}$ is the minor allele frequency of the i^th^ SNP.

The variance components estimated by AI-REML were used in the BLUPF90 module to predict GEBVs. The postGSf90 module was used to perform the wssGWAS. Marker effects and weights for constructing ***G*** are calculated in an iterative way as described by Wang et al., [6, 52]. Denote *t* as an iteration number and *i* as the *i*th SNP. The algorithm proceeds as follows:

1. Initialization, set t = 1, ${\mathbf{D}}_{(\mathrm{t})}=\mathrm{I}$, ${\mathbf{G}}_{\left(\mathrm{t}\right)}=\uplambda \mathbf{M}{\mathrm{D}}_{(\mathrm{t})}{\mathrm{M}}^{\mathrm{^{\prime}}}$ and $\uplambda = \frac{1}{{\sum }_{\mathrm{i}=1}^{\mathrm{m}}2{\mathrm{p}}_{\mathrm{i}}(1-{\mathrm{p}}_{\mathrm{i}})};$
2. Estimation of GEBV for all animals using ssGBLUP approach;
3. Computation of SNP effects as ${\widehat{u}}_{(\mathrm{t})}=\uplambda {\mathrm{D}}_{(\mathrm{t})}{\mathrm{M}}^{\mathrm{^{\prime}}}{\mathrm{G}}_{(\mathrm{t})}^{-1}{\widehat{a}}_{\mathrm{g}}$, where ${\widehat{u}}_{(\mathrm{t})}$ is a vector of SNP effects estimation and ${\widehat{a}}_{\mathrm{g}}$ is the GEBV of animals that were genotyped;
4. Calculation of SNP weights for the next iteration by ${\mathrm{d}}_{\mathrm{i}(\mathrm{t}=1)}={\widehat{\mathrm{u}}}_{\mathrm{i}\left(\mathrm{t}\right)}^{2}2{\mathrm{p}}_{\mathrm{i}}\left(1-{\mathrm{p}}_{\mathrm{i}}\right)$, where $\mathrm{i}$ is the ${\mathrm{i}}^{\mathrm{th}}$ SNP.
5. Normalization the SNP weights to keep the total genetic variance constant as ${\mathrm{D}}_{(\mathrm{t}+1)}=\frac{\mathrm{tr}({\mathrm{D}}_{\left(\mathrm{t}\right)})}{\mathrm{tr}({\mathrm{D}}_{\left(\mathrm{t}+1\right)})}{\mathrm{D}}_{(\mathrm{t}+1)}$;
6. $${\mathrm{G}}_{(\mathrm{t}+1)}= \frac{{\mathrm{MD}}_{(\mathrm{t}+1)}{\mathrm{M}}^{\mathrm{^{\prime}}}}{{\sum }_{\mathrm{i}=1}^{\mathrm{m}}2{\mathrm{p}}_{\mathrm{i}}(1-{\mathrm{p}}_{\mathrm{i}})};$$
7. Setting $\mathrm{t}=\mathrm{t}+1$ and loop to step 2.

The procedure was run for three iterations as used in Wang et al., [6], which reached a high accuracy of GEBVs. SNP effects obtained from the third iteration were used for calculating proportions of genetic variances explained by 0.5 Mb windows according to the linkage disequilibrium decay of this population [53]. The percentage of additive genetic variance explained by each window were calculated via

$$\frac{\mathrm{var}({\mathrm{a}}_{\mathrm{i}})}{{\upsigma }_{\mathrm{a}}^{2}}*100\mathrm{\%}=\frac{\mathrm{var}\left({\sum }_{\mathrm{j}=\mathrm{i}}^{\mathrm{x}}{\mathrm{z}}_{\mathrm{j}}{g}_{\mathrm{j}}\right)}{{\upsigma }_{\mathrm{a}}^{2}}*100\mathrm{\%}$$

where ${\mathrm{a}}_{\mathrm{i}}$ was the genetic value of the i-th region consisting of x = 0.5 Mb, ${\upsigma }_{\mathrm{a}}^{2}$ was the total genetic variance and ${\mathrm{z}}_{\mathrm{j}}$ was a vector genotype of the ${\mathrm{j}}^{\mathrm{th}}$ SNP for all animals; ${g}_{\mathrm{j}}$ is SNP effect of the ${\mathrm{j}}^{\mathrm{th}}$ SNP within the ${\mathrm{i}}^{\mathrm{th}}$ window. In this study, windows that explained > 1% of the additive genetic variance were highlighted as significant genomic regions associated with the analyzed traits.

### Identification of candidate genes and functional enrichment analysis

Based on the length of the genome covered by significant CNVRs and 0.5 Mb windows (windows that explained > 1% of the additive genetic variance) associated with LMA, LMD and LMP, the genes within these regions were searched on the Ensembl genome database version 99 of the *Sus scrofa* genome (Sscrofa11.1, http://jan2020.archive.ensembl.org) using the “biomaRt” package in R. The Kyoto Encyclopedia of Genes and Genomes (KEGG) and Gene Ontology (GO) enrichment analyses were conducted using KOBAS 3.0 [54]. Fisher’s exact test was used to assess the significance of the enriched terms with the criterion of *P* < 0.05 to explore the genes involved in pathways and biological processes. Furthermore, the GeneCards (http://www.genecards.org/) and NCBI (https://www.ncbi.nlm.nih.gov/) databases were used to query gene functions and determine promising candidates.

## Supplementary Information

**Additional file 1: Table S1.** Description of 695 CNVRs detected in the porcine genome.

**Additional file 2: Table S2.** Chromosome distribution of all 695 CNVRs detected in the porcine genome (based on* Sscrofa* 11.1 reference genome assembly).

**Additional file 3: Table S3.** Validation of CNVs detected in 50K SNP array using four different callers based on whole-genome resequencing data.

**Additional file 4: Table S4.** Descriptive statistics and variance components of growth traits in Duroc pigs.

**Additional file 5: Table S5.** The explained genetic variance of SNPs within significant windows for LMA.

**Additional file 6: Table S6.** The explained genetic variance of SNPs within significant windows for LMD.

**Additional file 7: Table S7. **The explained genetic variance of SNPs within significant windows for LMP.

**Additional file 8: Table S8.** Significant KEGG pathways and GO terms associated with growth traits. (*P* < 0.05).

**Additional file 9: Table S9.** Identified CNVRs compared with previous studies.

**Additional file 10: Table S10.** Read depth statistics and bin size selection for CNVnator.

**Additional file 11: Table S11. **qPCR primer and probe sequence information.

## Data Availability

The datasets of genotypes analyzed during the current study are available on figshare (https://doi.org/10.6084/m9.figshare.8019551.v1). The phenotypic data is not publicly available since the populations are consisted of the nucleus herd of Wens Foodstuff Group Co., Ltd., but are available from the corresponding author on reasonable request.

## Abbreviations

WssGWAS: Weighted single-step genome-wide association study
CNV: Copy number variation
CNVR: Copy number variation region
LMA: Loin muscle area
LMD: Loin muscle depth
LMP: Lean meat percentage
SNP: Single nucleotide polymorphisms
QTL: Quantitative trait locus
ssGBLUP: Single-step genomic best linear unbiased prediction
NGS: Next generation sequencing
qPCR: Real-time quantitative polymerase chain reaction
GO: Gene Ontology
KEGG: Kyoto Encyclopedia of Genes and Genomes
