# Supplementary material for: A composite strategy of genome-wide association study and copy number variation analysis for carcass traits in a Duroc pig population
Source: BMC Genomics. 2022 Aug 13;23:590. doi: 10.1186/s12864-022-08804-1 (PMC9375371; doi:10.1186/s12864-022-08804-1)
Supplement: Supplementary file 2 — Additional file 2: Table S2. Chromosome distribution of all 695 CNVRs detected in the porcine genome (based on Sscrofa 11.1 reference genome assembly). [file 12864_2022_8804_MOESM2_ESM.docx]

Table S2. Chromosome distribution of all 695 CNVRs detected in the porcine genome (based on *Sscrofa* 11.1 reference genome assembly).

| Chr | Chr length (bp) | CNVR count | Total CNVR length (bp) | Average size (bp) | Percentage (%) |
| --- | --- | --- | --- | --- | --- |
| 1 | 274330532 | 49 | 12710157 | 259390.96 | 4.63 |
| 2 | 151935994 | 49 | 18510025 | 377755.61 | 12.18 |
| 3 | 132848913 | 41 | 9793477 | 238865.29 | 7.37 |
| 4 | 130910915 | 50 | 11467631 | 229352.62 | 8.76 |
| 5 | 104526007 | 29 | 8008663 | 276160.79 | 7.66 |
| 6 | 170843587 | 54 | 14701327 | 272246.80 | 8.61 |
| 7 | 121844099 | 47 | 9881798 | 210251.02 | 8.11 |
| 8 | 138966237 | 39 | 7993095 | 204951.15 | 5.75 |
| 9 | 139512083 | 39 | 9559820 | 245123.59 | 6.85 |
| 10 | 69359453 | 15 | 3411920 | 227461.33 | 4.92 |
| 11 | 79169978 | 38 | 8709427 | 229195.45 | 11.00 |
| 12 | 61602749 | 34 | 9060699 | 266491.15 | 14.71 |
| 13 | 208334590 | 50 | 13263122 | 265262.44 | 6.37 |
| 14 | 141755446 | 55 | 12650097 | 230001.76 | 8.92 |
| 15 | 140412725 | 36 | 9182196 | 255061.00 | 6.54 |
| 16 | 79944280 | 29 | 5364159 | 184971.00 | 6.71 |
| 17 | 63494081 | 27 | 5432935 | 201219.81 | 8.56 |
| 18 | 55982971 | 14 | 4730413 | 337886.64 | 8.45 |
| Total | 2265774640 | 695 | 174430961 | 250979.80 | 7.70 |
