# Supplementary material for: A composite strategy of genome-wide association study and copy number variation analysis for carcass traits in a Duroc pig population
Source: BMC Genomics. 2022 Aug 13;23:590. doi: 10.1186/s12864-022-08804-1 (PMC9375371; doi:10.1186/s12864-022-08804-1)
Supplement: Supplementary file 4 — Additional file 4: Table S4. Descriptive statistics and variance components of growth traits in Duroc pigs. [file 12864_2022_8804_MOESM4_ESM.docx]

Table S4. Descriptive statistics and variance components of growth traits in Duroc pigs

| Traits | N | Genotyped | Mean (SD) | C.V(%) | $\sigma_{\alpha}^{2}$ | $\sigma_{\varepsilon}^{2}$ | $\sigma_{p}^{2}$ | *h*^2^ (SE) |
| --- | --- | --- | --- | --- | --- | --- | --- | --- |
| LMA (cm^2^) | 3941 | 3770 | 38.71 ± 3.55 | 9.17 | 4.44 | 4.80 | 9.24 | 0.48 (0.06) |
| LMD (mm) | 3941 | 3770 | 52.36 ± 3.72 | 7.1 | 5.48 | 7.38 | 12.86 | 0.43 (0.07) |
| LMP (%) | 3941 | 3770 | 62.23 ± 1.01 | 1.62 | 0.63 | 0.42 | 1.05 | 0.60 (0.07) |

N: sample size; C.V (%): coefficient of variation; LMP: lean meat percentage adjusted to 100kg; $\sigma_{a}^{2}$ = genetic variance, $\sigma_{e}^{2}$ = residual variance, $\sigma_{p}^{2}$ = phenotypic variance, $h^{2}$ = heritability.
